# Accelerated extracellular matrix turnover during exacerbations of COPD

**DOI:** 10.1186/s12931-015-0225-3

**Published:** 2015-06-11

**Authors:** Jannie M. B. Sand, Alan J. Knox, Peter Lange, Shu Sun, Jacob H. Kristensen, Diana J. Leeming, Morten A. Karsdal, Charlotte E. Bolton, Simon R. Johnson

**Affiliations:** Nordic Bioscience, Biomarkers and Research, Herlev Hovedgade 207, 2730 Herlev, Denmark; Section of Social Medicine, Institute of Public Health, University of Copenhagen, Oester Farimagsgade 5, 1014 Copenhagen K, Denmark; Division of Respiratory Medicine and Nottingham Respiratory Research Unit, University of Nottingham, Hucknall Road, Nottingham, NG5 1 PB UK; Section of Respiratory Medicine, Hvidovre Hospital, Kettegaards Alle 30, 2650 Hvidovre, Denmark

**Keywords:** COPD, Exacerbation, Extracellular matrix, Tissue turnover, Collagens, Elastin, Versican, Disease activity, Biomarkers

## Abstract

**Background:**

Exacerbations of chronic obstructive pulmonary disease (COPD) contribute significantly to disease progression. However, the effect on tissue structure and turnover is not well described. There is an urgent clinical need for biomarkers of disease activity associated with disease progression. Extracellular matrix (ECM) turnover reflects activity in tissues and consequently assessment of ECM turnover may serve as biomarkers of disease activity. We hypothesized that the turnover of lung ECM proteins were altered during exacerbations of COPD.

**Methods:**

69 patients with COPD hospitalised for an exacerbation were recruited at admission and returned for a 4 weeks follow-up. Competitive ELISAs measuring circulating protein fragments in serum or plasma assessed the formation and degradation of collagen types III (Pro-C3 and C3M, respectively), IV (P4NP 7S and C4M, respectively), and VI (Pro-C6 and C6M, respectively), and degradation of elastin (ELM7 and EL-NE) and versican (VCANM).

**Results:**

Circulating levels of C3M, C4M, C6M, ELM7, and EL-NE were elevated during an exacerbation of COPD as compared to follow-up (all *P* <0.0001), while VCANM levels were decreased (*P* <0.0001). Pro-C6 levels were decreased and P4NP 7S levels were elevated during exacerbation (*P* <0.0001). Pro-C3 levels were unchanged. At time of exacerbation, degradation/formation ratios were increased for collagen types III and VI and decreased for collagen type IV.

**Conclusions:**

Exacerbations of COPD resulted in elevated levels of circulating fragments of structural proteins, which may serve as markers of disease activity. This suggests that patients with COPD have accelerated ECM turnover during exacerbations which may be related to disease progression.

## Background

Chronic obstructive pulmonary disease (COPD) is a heterogeneous, slow progressing disease characterized by persistent airflow limitation resulting from chronic inflammation, structural changes, and small airway narrowing [1]. The main structural proteins of the extracellular matrix (ECM) of the lung are collagens, elastin, and proteoglycans. ECM turnover is part of healthy tissue maintenance, where old proteins are degraded and new proteins formed [2]. However, excessive ECM turnover may drive the structural changes in COPD and promote loss of lung function. A key challenge in COPD is the identification of biomarkers of disease progression [3]. ECM investigation by assessment of lung structural proteins may provide biomarkers of disease activity and prognosis. Novel technologies assessing ECM protein turnover are promising candidate tools [4, 5].

Exacerbations are periods of increased disease activity that drive COPD progression by accelerating loss of lung function [6], reducing quality of life [7], and causing mortality [8]. Patients in all COPD stages may experience exacerbations, although they become more frequent with increasing disease severity [9]. It is difficult to predict their occurrence, and the best predictor of future exacerbations is an exacerbation history [9, 10]. Although exacerbations are key events in COPD pathogenesis, little is known regarding structural changes in lung tissue during these events. Matrix metalloproteinase 9 (MMP-9) levels are known to be elevated while tissue inhibitor of metalloproteinase 1 (TIMP-1) levels are decreased in sputum of COPD patients at time of exacerbation compared to stable COPD [11], suggesting a destructive environment. In the present work we tested the hypothesis that ECM turnover, assessed systemically by biomarkers of protein degradation and formation, would be accelerated during an exacerbation of COPD where disease activity is high.

## Methods

### Study design

Patients presenting with a hospital admission deemed by a medical consultant to be a COPD exacerbation during 2011 and 2012 were recruited within 24 h of admission. All patients provided written informed consent, and the study was approved by the National Research Ethics Service (10/H0403/85). Blood samples were collected at time of exacerbation and at recovery: a 4 week follow-up visit performed a median of 30 (IQR 28–34) days after admission. At follow-up visit, the patients underwent standard post-bronchodilator spirometry, and performed a six minute walking distance (6MWD). Patient-reported measures included assessments of dyspnoea, using the Medical Research Council (MRC) dyspnoea scale, at follow-up visit, and smoking history. The inclusion criterion was a clinical diagnosis of acute COPD exacerbation at hospital admission made by a consultant physician. A physician diagnosis or radiological evidence of pneumonia was an exclusion criterion. The study comprised 69 patients with paired samples and with airflow obstruction (ratio of forced expiratory volume in one second (FEV_1_) to forced vital capacity (FVC) of <0.7) confirmed at follow-up visits.

### Biomarkers

Serum and plasma samples were stored at –80 °C until analysed. C3M, C4M, Pro-C3, P4NP 7S, ELM7, and EL-NE, were measured in serum, while C6M, Pro-C6, and VCANM were measured in plasma. All assays employed a monoclonal antibody directed against either a protein fragment produced by protease cleavage during degradation or formation, or an internal protein sequence. An overview of the assays used in this study and their technical specifications is given in Table 1. All samples were measured within the detection range of each assay and any sample with values below the lower limit of detection (LLOD) was assigned the value of LLOD.

**Table 1:** Overview of the assays used in this study to assess extracellular matrix turnover

Assay	Target	Detection range (ng/ml)	Intra-and inter-assay variation (%)	Reference level (ng/ml), mean (SD)	References
C3M	Collagen type III degraded by MMPs	5.52–177	3.4 and 9.8	15.3 (3.8)	[28]
C4M	Collagen type IV degraded by MMPs	22.8–748	4.2 and 18.5	55.4 (17.8)	[29]
C6M	Collagen type VI degraded by MMPs	4.88–420	8.0 and 11.0	8.85 (5.1)	[30]
ELM7	Elastin degraded by MMP-7	1.16–36.6	8.1 and 9.1	2.23 (0.74)	Preliminary data
EL-NE	Elastin degraded by neutrophil elastase	1.76–167	8.6 and 12.9	4.09 (2.24)	[31]
VCANM	Versican degraded by MMPs	0.78–7.13	3.0 and 7.6	1.20 (0.23)	[32]
Pro-C3	Collagen type III propeptide (N-terminal)	5.32–96.4	6.5 and 12.4	12.3 (4.4)	[33]
P4NP 7S	Collagen type IV 7S domain	32.9–3460	9.4 and 14.2	263 (91.3)	[34]
Pro-C6	Collagen type VI C5 domain	0.30–117	4.8 and 15.2	4.37 (0.69)	Preliminary data

The reference level was provided by the manufacturer (Nordic Bioscience) and refers to the biomarker level of a healthy population in the relevant matrix, *i.e.* serum (C3M, C4M, Pro-C3, P4NP 7S, ELM7, EL-NE) or plasma (C6M, Pro-C6, VCANM). SD, standard deviation; MMP, matrix metalloproteinase

### Statistical analysis

The main outcome predictor was change in circulating levels of C3M, C4M, C6M, Pro-C3, P4NP 7S, Pro-C6, ELM7, EL-NE, and VCANM. Based on previous data for these assays, for 90 % power to detect a 20 % difference in biomarker levels between exacerbation and follow-up with a two-sided α of 0.05, a minimum of 27 to 55 patients were required. Biomarker levels were normalised by log-transformation. Exacerbation and follow-up values were compared by paired *t*-test. Univariate and multivariate linear regressions were performed using the log-transformed biomarker data to identify associations with clinical parameters. The multivariate analysis included age, gender, body mass index (BMI), smoking pack years, and smoking status as additional explanatory variables. *P* <0.05 was considered significant. Analyses were performed using MedCalc Statistical Software v.12 (MedCalc Software, Ostend, Belgium).

## Results

### Basic demographics

Patient demographics and clinical characteristics are summarised in Table 2. Patients were mostly men (71 %) and ex-smokers (55 %). They were hospitalised for a median [IQR] of 3 [2–6] days, and follow-up visit was performed at 30 [28–34] days after admission.

**Table 2:** Basic characteristics of the COPD population at follow-up visit 4 weeks after exacerbation onset

Variable	Patients (*n* = 69)
Age (years), median (IQR)	67 (61–75)
Female sex, n (%)	20 (29)
BMI (kg/m^2^)	25.7 (6.3)
Current smokers, n (%)	31 (45)
Smoking pack years (years)	52 (26)
Length of hospitalisation (days), median (IQR)	3 (2–6)
FEV_1_ (liters)	1.19 (0.50)
FEV_1_ (% of predicted)	45.8 (16.1)
FVC (liters)	2.55 (0.81)
FVC (% of predicted)	77.5 (19.0)
FEV_1_/FVC ratio	0.46 (0.11)
6MWD (meters)	166 (119)
MRC dyspnoea score, median (IQR)	4 (3–4)

Variables are listed as mean (standard deviation) unless otherwise stated. IQR, interquartile range; BMI, body mass index; FEV_1_, forced expiratory volume in one second; FVC, forced vital capacity; 6MWD, 6 min walking distance; MRC, Medical Research Council

### Protein turnover during COPD exacerbation

**Fig. 1:**
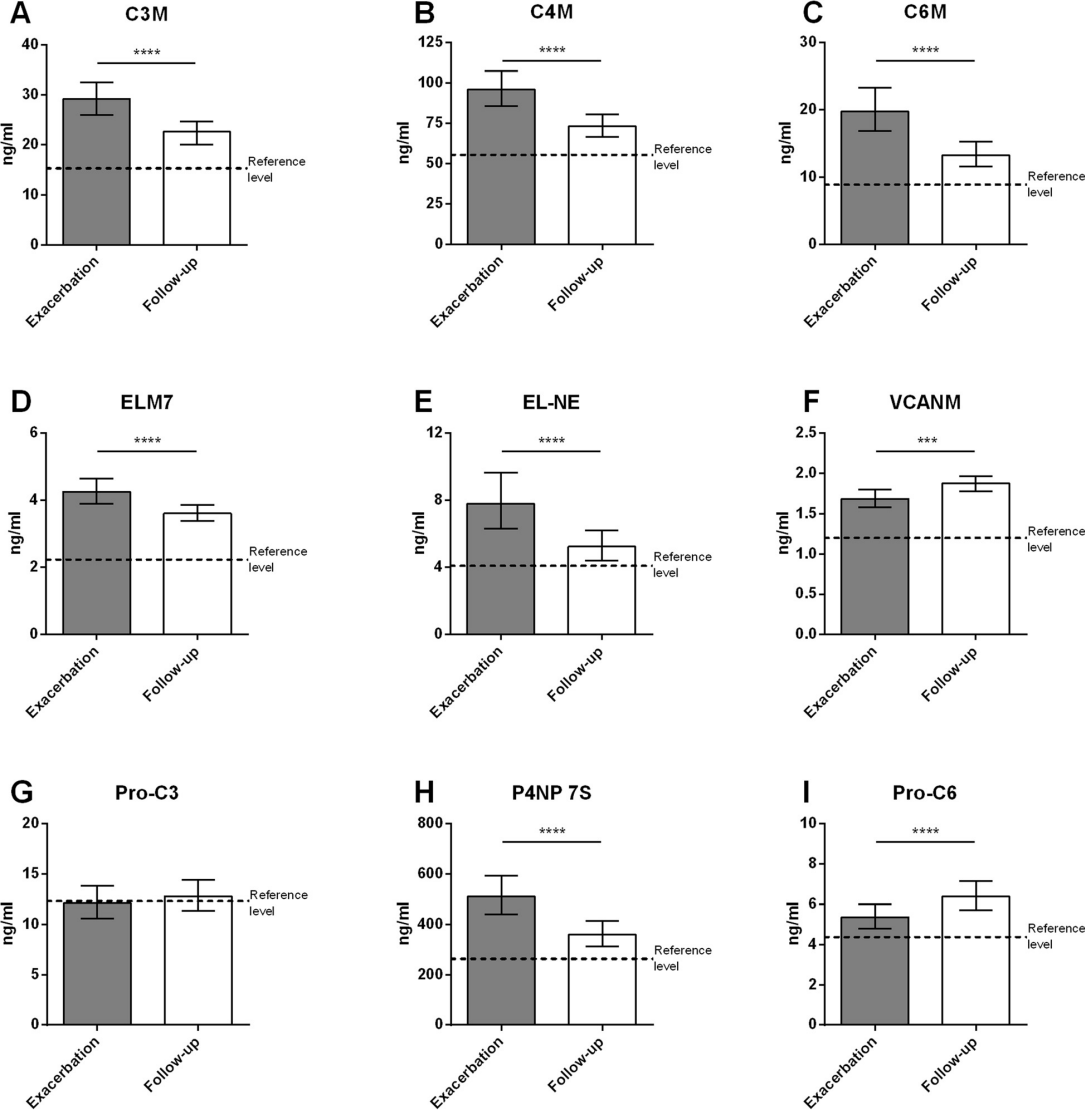
Biomarker levels at time of exacerbation and at 4 weeks follow-up. **a-f** Levels of degradation fragments of collagens type III (serum C3M), IV (serum C4M), and VI (plasma C6M), elastin (serum ELM7 and EL-NE) and versican (plasma VCANM). **g-i** Levels of fragments released in relation to formation of collagen types III (serum Pro-C3), IV (serum P4NP 7S), and VI (plasma Pro-C6). Results are shown as geometric mean with 95 % confidence intervals. Biomarker levels at exacerbation and follow-up were compared using paired *t*-test. Significance levels: ***P <0.001, ****P <0.0001

Circulating levels of protein fragments released at time of exacerbation and at a clinically stable disease period at 4 weeks follow-up are presented in Table 3. Degradation fragments of collagen type III (C3M), collagen type IV (C4M), collagen type VI (C6M), and elastin (ELM7 and EL-NE) were significantly elevated at exacerbation compared to follow-up (all *P* <0.0001; Fig. 1a-e). In contrast, a fragment of versican degradation (VCANM) showed a significantly decreased mean level at time of exacerbation (*P* <0.0001; Fig. 1f). Levels of fragments related to protein formation were not significantly changed for collagen type III (Pro-C3), but were increased for collagen type IV (P4NP 7S; *P* <0.0001) and decreased for collagen type VI (Pro-C6; *P* <0.0001) at exacerbation compared to follow-up (Fig. 1g-i). To investigate the effect of smoking on circulating levels of protein fragments, analysis was performed on current and ex smokers, individually, with similar results (data not shown).

**Table 3:** Biomarker levels at exacerbation and 4 weeks follow-up

	Exacerbation (ng/ml)	Follow-up (ng/ml)	*P* value
C3M	29.24 [26.32–32.49]	22.64 [20.78–24.67]	<0.0001
C4M	95.96 [85.83–107.28]	73.30 [66.59–80.69]	<0.0001
C6M	19.78 [16.82–23.27]	13.27 [11.56–15.23]	<0.0001
ELM7	4.26 [3.90–4.64]	3.61 [3.37–3.87]	<0.0001
EL-NE	7.79 [6.30–9.63]	5.23 [4.41–6.21]	<0.0001
VCANM	1.69 [1.58–1.80]	1.87 [1.78–1.97]	0.0001
Pro-C3	12.10 [10.60–13.81]	12.79 [11.35–14.42]	0.2549
P4NP 7S	510.99 [440.91–592.21]	359.20 [312.28–413.17]	<0.0001
Pro-C6	5.36 [4.81–5.99]	6.38 [5.71–7.14]	<0.0001

Results are presented as geometric mean [95 % confidence interval] and corresponding P values comparing circulating biomarker levels at time of exacerbation and follow-up

### Collagen degradation/formation balance

**Fig. 2:**
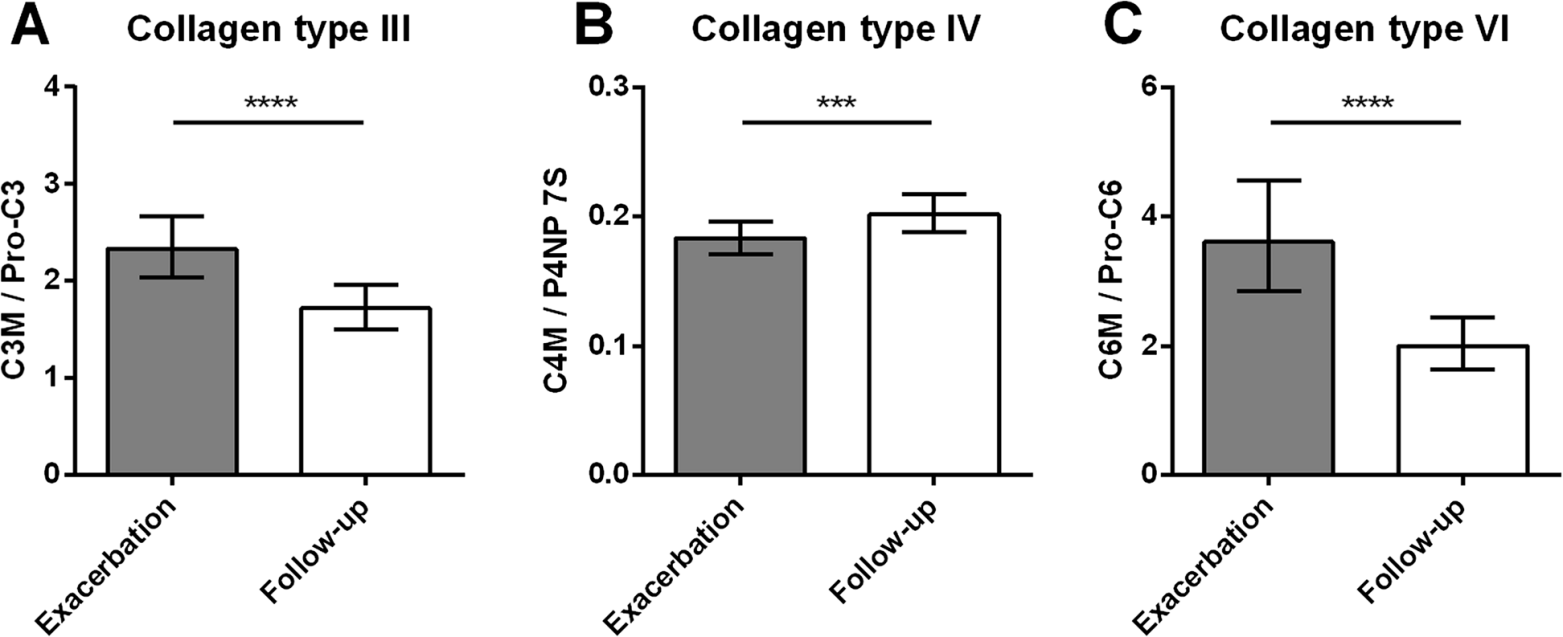
Ratios between collagen degradation and formation at time of exacerbation and at 4 weeks follow-up. Degradation/formation ratio of **a** collagen type III calculated by serum C3M/Pro-C3, **b** collagen type IV calculated by serum C4M/P4NP 7S, and **c** collagen type VI calculated by plasma C6M/Pro-C6. Results are shown as geometric mean with 95 % confidence intervals. Ratios at exacerbation and follow-up were compared using paired *t*-test. Significance levels: ***P <0.001, ****P <0.0001

The balance between degradation and formation of collagens was investigated by calculating the ratio between fragments of degradation and formation for collagen types III, IV, and VI (Fig. 2). The mean degradation/formation ratio [95 % CI] was significantly elevated at time of exacerbation for collagen type III (2.33 [2.03–2.66] vs. 1.72 [1.51–1.96], *P* <0.0001) and collagen type VI (3.61 [2.86–4.56] vs. 2.00 [1.64–2.44], *P* <0.0001). In contrast, the collagen type IV degradation/formation ratio was 0.18 [0.17–0.20] at exacerbation and increased to 0.20 [0.19–0.22] at follow-up (*P* = 0.0008).

### Associations with clinical parameters

At follow-up, C3M was positively associated with BMI (*r* = 0.245, *P* = 0.049), Pro-C6 was positively associated with age (*r* = 0.317, *P* = 0.022), and EL-NE was positively associated with white blood cell count (*r* = 0.279, *P* = 0.022). No associations were seen with smoking pack years, MRC score, length of hospitalisation, or sputum production. Pro-C3 levels were positively associated with FEV_1_ % of predicted value (%pred) and FVC %pred, and these remained significant after correcting for age, gender, BMI, smoking pack years, and smoking status (Table 4). 6MWD was negatively associated with C3M, C4M, C6M, and P4NP 7S (Table 4). Following correction for age, gender, BMI, smoking pack years, and smoking status, associations with C3M and C6M remained significant (Table 4).

**Table 4:** Associations between biomarker levels and clinical parameters

	FEV_1_ %pred	FVC %pred	6MWD
C3M	−0.118	−0.201	−0.350** (−0.311*)
C4M	−0.149	−0.161	−0.289* (−0.252)
C6M	−0.096	−0.226	−0.403** (−0.354**)
ELM7	−0.090	−0.149	−0.178
EL-NE	−0.039	−0.106	−0.186
VCANM	−0.0004	−0.080	−0.067
Pro-C3	0.345* (0.320*)	0.313* (0.305*)	0.055
P4NP 7S	−0.086	−0.193	−0.269* (−0.230)
Pro-C6	0.023	−0.046	−0.153

Results are presented as univariate correlation coefficients (r) for each marker. In brackets are given multivariate correlation coefficients for markers showing significant r. The multivariate linear regression analysis included age, gender, BMI, smoking pack years, and smoking status as additional explanatory variables. Significance levels: *P <0.05, **P <0.01. FEV_1_, forced expiratory volume in one second; %pred, percentage of predicted value; FVC, forced vital capacity; 6MWD, 6 min walking distance

## Discussion

To our knowledge, this is the first study describing that COPD exacerbations are associated with accelerated tissue turnover by measuring circulating levels of ECM protein fragments during and after an exacerbation. These results indicate that the processing of structural proteins in the lungs is altered during severe exacerbations as compared to a clinically stable disease period at 4 weeks follow-up. The main findings were; 1) significantly elevated levels of circulating biomarkers of collagen types III (C3M), IV (C4M), and VI (C6M), and elastin (ELM7 and EL-NE) degradation were found at time of exacerbation as compared to follow-up; 2) at exacerbation, levels of circulating biomarkers released in relation to protein formation was significantly elevated for type IV collagen, whereas it was significantly decreased for type VI collagen, and unchanged for type III collagen; 3) the tissue turnover balance, assessed systemically by the collagen degradation/formation ratio, was increased for type III and VI collagen and decreased for type IV collagen; 4) circulating levels of Pro-C3 were associated with disease severity, assessed by FEV_1_ %pred and FVC %pred, while C3M, C4M, C6M, and P4NP 7S were associated with exercise capacity, assessed by 6MWD.

Structural ECM proteins are crucial for maintaining tissue function. Collagens provide flexibility and form a scaffold for cells, elastin provides tissue elasticity, and proteoglycans such as versican maintain ECM integrity by interacting with hyaluronan. Others have investigated the collagen turnover by different technologies. O’Reilly *et al.* reported elevated sputum proline-glycine-proline (PGP), a neutrophil chemoattractant derived from collagen degradation, around the time of exacerbation in a small cohort of COPD patients [12]. Further, azithromycin treatment targeting exacerbations reduced sputum PGP. Their results indicate that COPD exacerbation causes collagen destruction, resulting in PGP release. As the PGP motif exists in several ECM proteins, especially collagens [13], and its sputum levels reflect overall lung ECM degradation; we investigated ECM turnover further by analysing different proteins. Further, our study utilised samples from the same patients 4 weeks apart, providing insight into short-term changes in protein turnover. We previously reported elevated biomarker levels, including serum C3M and C6M, in a small cohort of mild COPD patients compared to healthy controls [5]. Our previous study utilised early research and development versions of some of the assays presented here, resulting in somewhat different levels, but nevertheless indicating that measuring protein fragments in the circulation of patients with COPD may be of interest.

Collagens are the most abundant components of the lung interstitium, and fibrillar collagens such as types I and III maintain the lung architecture. Collagen type IV is the main constituent of the basement membrane, a sheet-like ECM structure underlying epithelial and endothelial cells. Collagen type VI molecules form anchoring fibrils that can interact with collagen types I and IV and fibronectin thereby adhering to the interstitial matrix and basement membrane, upholding tissue structure [14]. Our results indicate that both the interstitial matrix and the basement membrane were turned over faster during an exacerbation and point to a destructive environment affecting several structural proteins. Both formation and degradation of collagen types III, IV, and VI were changed. Significantly decreased plasma Pro-C6 levels during a COPD exacerbation suggest that collagen type VI formation was lower than during stable disease. Consistent with this, the collagen type VI degradation/formation ratio was significantly elevated during exacerbation when compared to follow-up, indicating a balance shift towards collagen type VI degradation during exacerbation. As collagen type VI is involved in reconstructing damaged tissue by stimulating fibroblast proliferation and preventing apoptosis [15–17], its enhanced degradation during exacerbation may contribute to COPD progression. It should be noted that the process of proteins and peptides entering the circulation is not fully understood. Different peptides may diffuse from the lung tissue to the circulation in different manners. Thus, the degradation/formation ratios cannot be interpreted directly but may serve as an indication of the shift in the balance between degradation and formation at time of exacerbation and follow-up. The ratio as such does not provide any information on the degree of protein formation and degradation. Collagen type III formation was not affected by exacerbation as shown by unaltered levels of serum Pro-C3. However, degradation (serum C3M) and degradation/formation ratio were significantly elevated during exacerbation, suggesting an overall collagen type III destruction during these episodes. As collagen type III is an abundant lung ECM protein, its destruction may greatly alter tissue function. Interestingly, serum Pro-C3 was the only biomarker directly associated with FEV_1_ %pred, indicating a relation to disease severity rather than activity. Levels of serum C4M and P4NP 7S were significantly increased during exacerbation compared to follow-up. This indicated that both formation and degradation of collagen type IV were elevated during exacerbation, resulting in an accelerated turnover rate. The balanced outcome of this turnover showed a decreased degradation during exacerbation compared to follow-up, as shown by the C4M/P4NP 7S ratio. C3M, C4M, C6M, and P4NP 7S were negatively associated with 6MWD, indicating an interesting relationship between exercise capacity and collagen turnover that should be investigated further.

Elastin degradation by MMP-7 and neutrophil elastase was increased during exacerbation, as shown by elevated levels of serum ELM7 and EL-NE, respectively. Elastin provides lungs with elasticity, allowing movement during breathing and preservation of the lung structure and function. Neutrophil elastase is released by neutrophils which are abundant during tissue inflammation [18], and degradation of elastin by neutrophil elastase (EL-NE) may reflect the local inflammatory response. Neutrophil elastase activity towards fibrinogen and the proportion of blood neutrophils are elevated during a COPD exacerbation compared to stable disease [19, 20], demonstrating a role of neutrophil elastase in exacerbations. Our data support this finding by showing increased activity of neutrophil elastase towards elastin at time of exacerbation. Although our results for ELM7 and EL-NE were similar, it is important to recognize the difference in the assays and the value of knowing exactly what epitope the antibody targets [21].

Interestingly, plasma VCANM was decreased during exacerbation, implying that versican processing is different to other ECM proteins. Versican degradation is linked to vascular diseases, suggesting that proteolytic modifications have pathophysiological implications for the vascular system [22]. However, versican also seems to play an important role in the lungs and versican content in the lungs is increased in COPD patients compared to controls [23, 24]. The increased versican content is believed to inhibit the function of elastin binding protein (EBP), thus impairing formation of elastic fibers [25]. An increased versican content in the lungs was associated with decreased elastin and EBP content and a decrease in FEV_1_ [23] supporting a different role for versican compared with elastin and collagens in COPD.

Exacerbations of COPD are periods of increased disease activity, when the biological processes that drive disease progression may be accelerated [26]. While FEV_1_ is a measure of disease severity it is not a good measure of disease activity, and a low FEV_1_ does not necessarily reflect a fast disease progression [3]. The rate of decline in FEV_1_ represents a better measure of disease activity or disease progression [3, 27]. However, measuring the rate of decline in FEV_1_ requires very large and long-term studies, making it impractical for assessment of new disease-modifying treatments [3]. The recent ATS/ERS statement on research questions in COPD proposes measures of lung tissue destruction as potential biomarkers of disease activity and recommends further studies on disease activity biomarkers [26]. The results presented here indicate that biomarkers of lung ECM turnover may be markers of disease activity as circulating levels are elevated during exacerbation. These markers assess protease-generated protein fragments, also known as protein fingerprints [21], derived from tissue. The assays used are very sensitive, allowing measurements of low abundance protein fragments in circulation. This provides an indirect measure of tissue turnover in the lung, which may reflect disease activity. This assessment may better reflect dynamic remodelling of lung tissue compared to spirometry measures, which in the stable state reflect the cumulative disease process and reflect both the baseline level and the additional bronchoconstriction during the acute exacerbation. Future studies on the ECM turnover in patients classified as slow or fast progressors may reveal if patients with higher disease activity have elevated biomarker levels also in the stable state. Reliable markers of disease activity are an urgent need in the field of COPD as they have the potential to improve and shorten the assessment of new treatments [3].

A limitation to this study was that biomarkers assessed systemically may originate from multiple organs, thus introducing a background level from other tissues. However, the turnover in healthy tissue is balanced and occurs at a slow pace with release of low levels of protein fragments. In this study we compared circulating levels in a patient group at two time points, minimising the risk that observed differences are not the cause of the exacerbation. Future studies should assess the ECM turnover in sputum for comparison, eliminating the extra-pulmonary contribution. Another limitation was that treatment for exacerbation may influence protein turnover. However, irrespective of treatment this study showed accelerated ECM turnover during exacerbation. This study represents daily clinical practice for COPD patients admitted to hospital for an exacerbation, thus it includes patients on different treatment regimens and with exacerbation from different causes. Further, co-morbidities may influence biomarker levels and the lack of information on these is a limitation, however the paired design of this study may eliminate some of the effect. Although patients did not have evidence of myocardial infarction or other significant events during hospitalisation, we cannot exclude that subclinical events may have occurred affecting the levels of the measured biomarkers. Future studies should explore the effect of treatment and co-morbidities on ECM turnover as well as exacerbation aetiology.

## Conclusions

We have shown that during an acute exacerbation of COPD there is an accelerated turnover of ECM proteins relevant to lung. The enhanced protein turnover is likely to be important in COPD progression. Assessment of these biomarkers may provide useful endpoints in proof-of-concept studies aiming at reducing lung tissue turnover during exacerbations.

## Abbreviations

%pred: Percentage of predicted value
6MWD: Six minutes walking distance
BMI: Body mass index
CI: Confidence interval
COPD: Chronic obstructive pulmonary disease
EBP: Elastin binding protein
ECM: Extracellular matrix
FEV_1_: Forced expiratory volume in one second
FVC: Forced vital capacity
IQR: Interquartile range
LLOD: Lower limit of detection
MMP: Matrix metalloproteinase
MRC: Medical research council
PGP: Proline-glycine-proline
SD: Standard deviation
TIMP: Tissue inhibitor of metalloproteinase
